# Cybrid Model Supports Mitochondrial Genetic Effect on Pig Litter Size

**DOI:** 10.3389/fgene.2020.579382

**Published:** 2020-12-15

**Authors:** Hao Liu, Jikun Wang, Dan Wang, Minghua Kong, Chao Ning, Xing Zhang, Jinlong Xiao, Xin Zhang, Jianfeng Liu, Xingbo Zhao

**Affiliations:** ^1^College of Animal Science and Technology, China Agricultural University, Beijing, China; ^2^Wenshang Professor Workstation of China Agricultural University, Jining, China; ^3^Key Laboratory of Qinghai-Tibetan Plateau Animal Genetic Resource Reservation and Utilization, Southwest Minzu University, Chengdu, China; ^4^College of Animal Science and Veterinary Medicine, Shandong Agricultural University, Taian, China; ^5^Jining Animal Husbandry Station, Jining, China

**Keywords:** mitochondrial DNA, genetic effect, cybrid, litter size, pig

## Abstract

In pigs, mitochondrial DNA (mtDNA) polymorphism and the correlation to reproductive performance across breeds and individuals have been largely reported, however, experimental proof has never been provided. In this study, we analyzed 807 sows for correlation of total number born (TNB) and mitotype, which presented the maximum of 1.73 piglets for mtDNA contribution. Cybrid models representing different mitotypes were generated for identification of the mtDNA effect. Results indicated significant differences on cellular and molecular characteristics among cybrids, including energy metabolic traits, mtDNA copy numbers and transcriptions, mRNA and protein expressions on mitochondrial biogenesis genes and reproduction-related genes. Referring to mitotypes, the cybrids with prolific mitotypes presented significantly higher oxygen consumption rate (OCR) productions, mtDNA transcriptions and copy numbers than those with common mitotypes, while both mRNA and protein expressions of *PPARA*, *TFAM*, *ER1*, *ER2*, and *ESRRG* in prolific cybrids were significantly higher than those with common mitotypes. Cybrid models reflected the mtDNA effect on pig litter size, suggesting the potential application of mtDNA polymorphism in pig selection and breeding practices.

## Introduction

The mitochondrion is indispensable to eukaryotes, which involves in bioenergy metabolism, biosynthesis, anti-oxidant defense, signal transduction, redox status, and apoptosis (Wallace, 2012; Aanen et al., 2014; Wang et al., 2017b), hence the mitochondrial dysfunction may result in the decline of individual motor skill or even disease (Amaral et al., 2013; Cardoso et al., 2017; Herst et al., 2017; van der Bliek et al., 2017). Mitochondrial genome (mtDNA) is necessary for components of the respiratory chain complexes, which is essential for ATP production. In vertebrates, mtDNAs are approximately 16.5 kb-circular molecules, of which encodes 37 canonical genes, including 2 rRNAs, 22 tRNAs, and 13 polypeptide genes for respiratory chain subunits. Mitochondrial genome has been widely used to study origin evolution (Xiang et al., 2014), diseases (Wallace, 1994), aging (Boengler et al., 2017), and production and reproduction traits of farm animals (Reicher et al., 2012; Liu et al., 2019a).

The reproductive performance is critical important for pig production, which has been reported on the mtDNA effect. Previous investigations illustrated significant differences for porcine oocyte maturation (Tsai et al., 2016) and oocyte number (Liu et al., 2019a), fertilization (Tsai et al., 2016), litter size (Wang et al., 2018), and teat quality (St John and Tsai, 2018) amongst mtDNA haplotypes (mitotypes) or mtDNA haplogroups (mitogroups).

However, the mtDNA effect on pig reproduction traits lacks the experimental proof. In this study, we focused on the litter size, the core trait for reproduction performance, to investigate the relationship with mitotypes, and using transmitochondrial cell model (cybrid) to test the validity of mtDNA effects.

## Materials and Methods

### Ethics Statement

The guidelines of the experimental animal management of China Agricultural University (CAU) were followed throughout the study, and the experimental protocols were approved by the Experimental Animal Care and Use Committee of CAU.

### Sample Collection and Association Analysis

The records of TNB were collected and amounted to 1,766 in 807 Large White sows from Beijing Liuma pig company. All pigs used in this study lived in consistent circumstances (including farm, feeding and management, etc.). Ear samples were collected, and genomic DNAs were extracted using a Tissue/Cell Genome DNA Extraction Kit (Aidlab, Beijing, China) according to manufacturer’s instruction. Mitochondrial genome was amplified in a 50 μL volume containing 25 μL 2 × A9 LongHiFi PCR MasterMix (Aidlab, Beijing, China), 1 μL each forward and reverse primers (referred to NC_000845.1, Supplementary Table 1), 2 μL DNA and 21 μL H_2_O using the following program: 95°C for 3 min, followed by 38 cycles of 95°C for 10 s, 60°C for 15 s and 72°C for 3 min, and PCR products were directly sequenced by TsingKe Biotech (China).

Mitochondrial DNA polymorphic sites were detected through sequence alignment using MEGA7 software^1^. Mitotypes were sorted by FaBox online software^2^ and analyzed through median joining method by Network 5 software^3^, and mitogroups were sorted according to mutated positions among mitotypes. Association analyses of mitotype/mitogroup and TNB were performed by the linear mixed model using ASReml software as follows:

TNB=μ+ys+parity+variation+ID+EP+e

In the model, the effects of pig population mean (μ), farrowing year-season (***ys***), parity number (***parity***), mtDNA variation (including mitotypes and mitogroups), the polygenic effect (***ID***), the permanent environmental effect (***EP***), and the random residual (***e***) were included. The response variable was TNB. The polygenic effect corrected the genetic background by the additive genetic relationship matrix, i.e., the pedigree information. The permanent environmental effect dealt with the repeated measurement data. When a set of statistical inferences were simultaneously considered, multiple comparisons were conducted by the Bonferroni method, and the adjusted *P* < 0.05 was regarded as statistical significance.

### Cybrid Generation and Culture

Cybrids were generated by enucleation of mitochondria donor cells and fusion of the cytoplasts with ρ0 cells according to modified procedures of the previous study (Yu et al., 2015; Wang et al., 2017a). Ear primary fibroblasts were used as mitochondria donors, which were isolated from Large White sows using tissue culture method (Wang et al., 2017a). And IPEC-J2 cell line was used as nucleus donor (ρ0 cell), which was presented by Dr. Jianguo Zhao, Chinese Academy of Sciences, China. Briefly, IPEC-J2 was cultured in former medium (containing 10% FBS, 4 μg/ml rhodamine 6G, 50 μg/mL uridine, 1 mM sodium pyruvate and 1% penicillin-streptomycin; Gibco, United States) for 7 days with replacement of former medium at 24 h intervals to generate ρ0 cells. Before cytoplast fusion with fibroblasts, ρ0 cells were cultured in normal medium for 3 h. Primary fibroblasts were enucleated using ultracentrifugation (44,000 g) with cytochalasin B. Cybrids were created by the fusion of the ρ0 cells with the enucleated cells using polyethylene glycol (PEG), and positive cells were identified by mtDNA sequencing.

Fibroblasts and cybrids were cultured in DMEM (Gibco, United States) supplemented with 10% FBS (Gibco, United States), 100 units/ml penicillin and 100 μg/ml streptomycin (Gibco, United States), at 37°C with 5% CO_2_.

### Cell Proliferation Assay

An Enhanced Cell Counting Kit-8 (CCK-8, Beyotime, China) was used in the cell proliferation assay, wherein 3 × 10^3^ fibroblasts/well or 2 × 10^3^ cybrids/well were seeded in 96-well plates, respectively. After 1–7 days culture, cells were further incubated with 10 μL of CCK-8 solution for 2 h at 37°C, respectively. Then, the absorbance at 450 nm was measured with reference wavelength at 650 nm using a microplate reader.

### Analysis of Mitochondrial Respiration Ability

Approximately 1.8 × 10^4^ cells for fibroblasts and 8 × 10^3^ cells for cybrids were seeded in 96 wells of XF96 cell culture microplates (Seahorse Bioscience, United States), respectively. For respiratory analysis, cells were analyzed according to the procedures described in the Seahorse XF Cell Mito Stress Test kit (Agilent, United States). After baseline measurements, OCR values were measured after sequentially adding Oligomycin (2 μM final concentration), FCCP (carbonyl cyanide 4-trifluoromethoxy-phenylhydrazone, 0.5 μM final concentration for fibroblasts and 1 μM for cybrids), and Rotenone plus Antimycin A (0.5 μM final concentration of each). Subtracting the non-mitochondrial OCR from the total OCR yields the mitochondrial OCR.

### Quantification of mtDNA Copy Number

Genomic DNAs were extracted using a Tissue/Cell Genome DNA Extraction Kit (Aidlab, Beijing, China). The mtDNA specific primers were listed in Supplementary Table 1 were designed using GenBank sequence (NC_000845.1). *Beta-globin* gene was used as the internal standard (Liu et al., 2019b). The mtDNA copy number of each sample was compared by calculating the ratio of mitochondrial to nuclear DNA abundance (mtDNA/nDNA) (Wang et al., 2017a). The baseline adjustment method of the qPCRsoft software (ANALYTIKJENA, Germany) was used to determine the Ct of each reaction. The amplification efficiencies were close to 100%, and each RT-qPCR experiment was performed in triplicates.

### The mRNA Expression Levels of Mitochondrial H Strand, Mitochondrial Biogenesis-Related Genes, and Reproduction-Related Genes

RNA samples of primary fibroblasts and cybrids were extracted by Tissue/Cell RNA Rapid Extraction Kit (Aidlab, China) according to the manufacturer’s instruction, and the cDNA was synthesized using 1 μg RNA with TRUE script One Step RT-PCR Kit (Aidlab, China). The relative expressions of mitochondrial H strand, as well as 11 genes (*NRF-1*, *PPARA*, *TFAM*, *TFB1M*, *TFB2M*, *PPARGC1A*, *ER1*, *ER2*, *ESRRA*, *ESRRG*, and *FSHR*) were measured by RT-qPCR with specific primers (Supplementary Table 1). *GAPDH* was used as the internal control (Li et al., 2016b). The baseline adjustment method of the qPCR software (ANALYTIKJENA, Germany) was used to determine the Ct of each reaction. The amplification efficiencies were close to 100%, and all samples were amplified in triplicate. For data analysis, 2^–ΔΔct^ method was used to calculate the relative level of samples.

### Western Blotting

The protein levels of five genes (*TFAM*, *PPARA*, *ER1*, *ER2*, and *ESRRG*) were measured by Western blotting. Protein samples were extracted using Cell lysis buffer for Western and IP (Beyotime, China). Samples were transferred to polyvinylidene fluoride membrane (Solarbio, China), which were then blocked for 2 h in Western Blocking Buffer (Beyotime, China) at 37°C. Membranes were incubated overnight at 4°C with the rabbit polyclonal antibodies against TFAM (1: 1,000), PPARA (1: 1,000), ER1 (1: 500), ER2 (1: 500), ESRRG (1: 1,000, Bioss, China) or a monoclonal antibody against GAPDH (1: 1,000, Beyotime, China), respectively. Then, membranes were washed and incubated with HRP labeled goat anti-rabbit IgG (1: 1,000, Beyotime, China) for 1 h. Next, proteins on the membranes that reacted with the antibodies were visualized using a DAB Horseradish Peroxidase Color Development Kit (Beyotime, China). Signals were captured through film coloration and measured using ImageJ software (NIH, United States).

### Reactive Oxygen Species (ROS) Detection

The ROS level was determined by ROS Assay Kit (Beyotime, China). Approximately 4 × 10^5^ cells in a 6-well plate were incubated with DCFH-DA (10 mM) for 20 min at 37°C and washed 3 times with DMEM. ROS production in cells was measured fluorometrically with excitation and emission settings at 488 and 525 nm, respectively, and expressed as arbitrary units.

### Statistical Analysis

The mean values shown in Figures 2–6 and used in the statistical analyses represented at least three independent trials. Differences between each group were evaluated by analysis of variance (ANOVA), followed by Duncan’s multiple-range test using the General Linear Model procedure of SAS (SAS version 8.2; Cary, NC, United States). Results were expressed as the mean ± standard error of the mean (SEM), and the data used in the statistical analyses represented at least three independent trials. Results with *P*-values < 0.05 or < 0.01 were considered statistically significant or extremely significant when testing for differences among samples, respectively.

## Results

### Association Analysis of mtDNA Variants and TNBs

**FIGURE 1:**
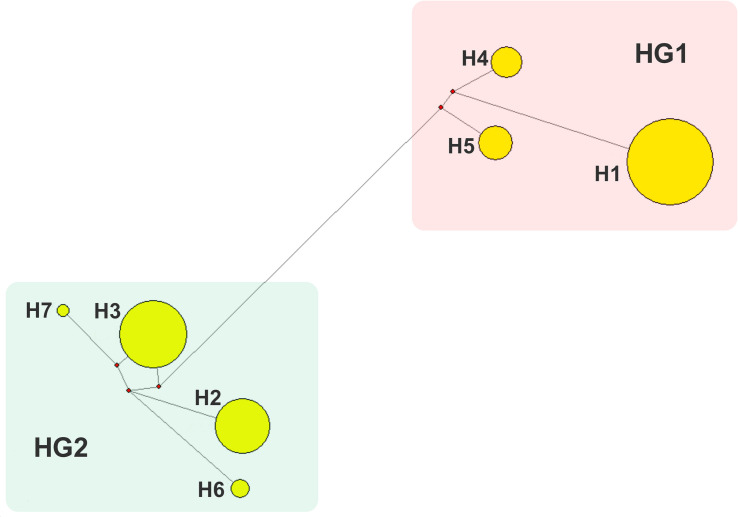
Network analysis of mitotypes. 7 mitotypes clustered into two mitogroups, HG1 (H1, H4, and H5) and HG2 (H2, H3, H6, and H7).

Totally 220 polymorphic sites of mitochondrial genome were detected among 807 sows, which assigned into 7 mitotypes (H1–H7, Supplementary Table 2), and clustered into 2 mitogroups (HG1 and HG2, Figure 1). Among them, 179 mutation sites were completely linked and formed the basis of two mitogroups (HG1 and HG2, Supplementary Table 3). HG1 included H1, H4 and H5, and HG2 contained mitotype H2, H3, H6, and H7 (Figure 1). Mitogroups were significantly associated with TNBs (*P* < 0.01), and the difference reached 1.07 piglets (Table 2). For mitotypes, TNBs with H1, H2, H4, and H5 were significantly higher than those of H3, and H1 and H4 owned larger TNBs compared to H6 (*P* < 0.05, Table 1). Notably, the largest difference of TNBs was between H4 and H3, which reached 1.73 piglets (*P* < 0.05, Table 1).

**TABLE 1:** Association analysis of TNBs and mitotypes.

**Mitotype**	**Sow number**	**TNB**	
		**Record number**	**LSMEAN ± SEM**
H1	341	747	11.948 ± 0.324^a^
H2	134	299	11.626 ± 0.369^a,b^
H3	210	480	10.467 ± 0.339^c^
H4	43	82	12.192 ± 0.514^a^
H5	56	103	11.823 ± 0.471^a,b^
H6	15	34	10.511 ± 0.762^b,c^
H7	8	21	11.247 ± 0.973^a,b,c^

*TNB, total number born; LSMEAN, least square means; SEM, standard error of the mean. Different letters by superscript mean significant difference at P < 0.05 level.*

**TABLE 2:** Association analysis of TNBs and mitogroups.

**Mitogroup**	**Sow number**	**LSMEAN ± SEM**	***P*-value**
HG1	440	12.003 ± 0.320	<0.05
HG2	367	10.938 ± 0.320	

*TNB, total number born; LSMEAN, least square means; SEM, standard error of the mean.*

### Oxygen Consumption Rate and ROS Assay

Ear primary fibroblasts (named PH1-PH7) were isolated from pigs with mitotypes of H1–H7, respectively (3 repeats for each mitotype), which were used as mitochondrial donors for generating cybrids (named IPH1-IPH7, respectively).

**FIGURE 2:**
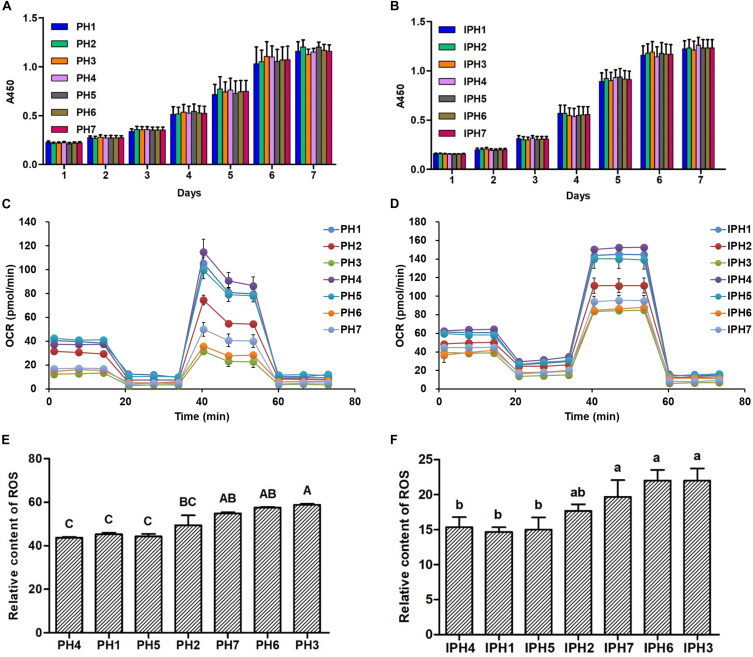
Cell proliferation, oxygen consumption rate and ROS assays in different cell lines. **(A)** Cell proliferation in fibroblasts. **(B)** Cell proliferation in cybrids. **(C)** OCR assays for fibroblasts. **(D)** OCR assays for cybrids. **(E)** The relative ROS contents in fibroblasts. **(F)** The relative ROS contents in cybrids. Different capital or lowercase letters meant significant differences at *P* < 0.01 or *P* < 0.05, respectively. PH1-7, fibroblast with mitotype 1–7, respectively; IPH1-7, cybrid with mitotype 1–7, respectively.

The cell proliferation exhibited similar levels at different detecting days among primary fibroblasts or cybrids (Figures 2A,B, *P* > 0.05). Oxidative phosphorylation (OXPHOS) properties for fibroblasts and cybrids were illustrated in Figures 2C,D. For fibroblasts, PH1, PH4, and PH5 exhibited statistically higher OCR indexes (basal respiration, ATP production, maximal respiration and spare respiratory capacity) than those of PH2, PH3, PH6, and PH7 (*P* < 0.01), and the levels of basal respiration and maximal respiration in PH2 were significantly higher than PH3, PH6, and PH7 (*P* < 0.01), and PH3 and PH6 had lower maximal respiration and spare respiratory capacity compared to PH2 (*P* < 0.01) (details in Supplementary Figures 1A–D). Similar results appeared in cybrids: IPH1, IPH4, and IPH5 presented greater OCR indexes (basal respiration, ATP production, maximal respiration and spare respiratory capacity) than IPH2, IPH3, IPH6, and IPH7 (*P* < 0.01), and IPH2 exhibited higher basal respiration than IPH3, greater maximal respiration than IPH3, IPH6 and IPH7, and stronger spare respiratory capacity than IPH3 and IPH6 (*P* < 0.01) (details in Supplementary Figures 1E–H).

Meanwhile, ROS content was also measured among fibroblasts or cybrids. For primary cells, PH3 reached the higher level than PH1, PH2, PH4, and PH5 (Figure 2E, *P* < 0.01), and PH6 and PH7 produced more ROS than PH1, PH4, and PH5 (*P* < 0.01). For cybrids, IPH3, IPH6, and IPH7 exhibited significantly higher levels than IPH1, IPH4, and IPH5 (Figure 2F, *P* < 0.05).

### mtDNA Copy Numbers and Transcriptions

**FIGURE 3:**
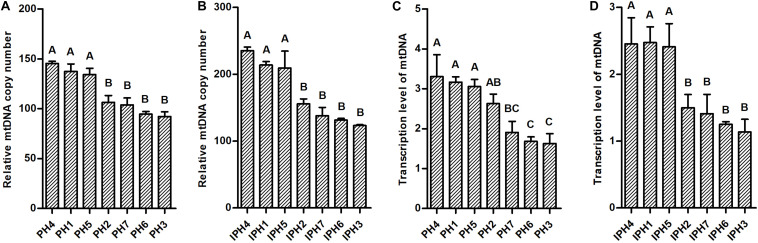
Detections mitochondrial DNA copy number and transcription in fibroblasts and cybrids by RT-qPCR assays. **(A)** mtDNA copy numbers in fibroblasts. **(B)** mtDNA copy numbers in cybrids. **(C)** Transcription levels of mtDNAs in fibroblasts. **(D)** Transcription levels of mtDNAs in cybrids. Different letters on columns meant extremely significant differences at *P* < 0.01. PH1-7, fibroblast with mitotype 1–7, respectively, IPH1-7, cybrid with mitotype 1–7, respectively.

Mitochondrial DNA copy numbers and transcription levels in fibroblasts and cybrids were detected using RT-qPCR assays. PH1, PH4, and PH5 owned significantly larger abundance of mtDNA copies than PH2, PH3, PH6, and PH7 (Figure 3A, *P* < 0.01), while, IPH1, IPH4, and IPH5 harbored more mitogenomes than other cybrids (Figure 3B, *P* < 0.01). For transcription levels, PH4, PH1, and PH5 showed higher expressions than PH3, PH6, and PH7 (Figure 3C, *P* < 0.01), and PH2 produced more abundant mtDNA transcripts than PH6 and PH3 (*P* < 0.01), respectively. For cybrids, IPH2, IPH3, IPH6, and IPH7 exhibited lower levels than IPH1, IPH4, and IPH5 (Figure 3D, *P* < 0.01).

### Expression Levels of Mitochondrial Biogenesis-Related Genes

**FIGURE 4:**
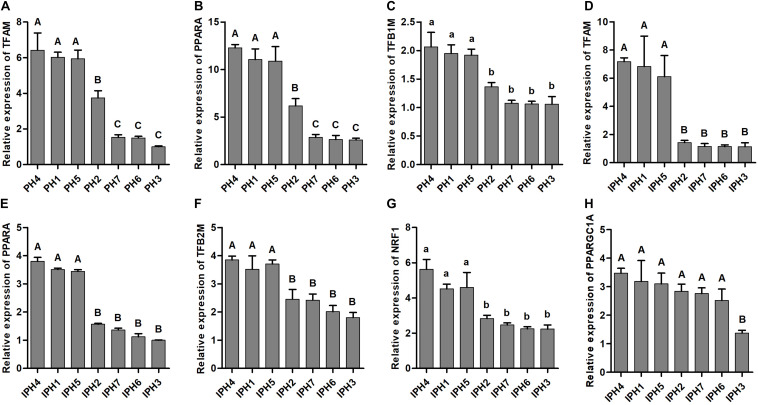
mRNA expressions of mitochondrial biogenesis-related genes in fibroblasts and cybrids. **(A–C)** Illustrated the expressions of *TFAM*, *TFB1M*, and *PPARA* in fibroblasts, **(D–H)** presented the expressions of *TFAM*, *TFB2M*, *PPARA*, *NRF1*, and *PPARGC1A* in cybrids. Capital or lowercase letters on columns meant significant differences at *P* < 0.01 or *P* < 0.05 levels, respectively. PH1-7, fibroblast with mitotype 1–7, respectively; IPH1-7, cybrid with mitotype 1–7, respectively.

Six mitochondrial biogenesis-related genes (*TFAM*, *TFB1M*, *TFB2M*, *PPARA*, *NRF1*, and *PPARGC1A*) were measured for expression abundance in fibroblasts and cybrids (Figure 4). PH1, PH4, and PH5 possessed larger expression abundances of *TFAM*, *TFB1M*, and *PPARA* than PH2, PH3, PH6, and PH7 (Figures 4A–C, *P* < 0.05), and PH2 owned higher levels of *TFAM* and *PPARA* compared to PH3, PH6 and PH7 (*P* < 0.01). For cybrids, IPH1, IPH4, and IPH5 harbored significantly higher levels of *TFAM*, *TFB2M*, *PPARA*, and *NRF1* than IPH2, IPH3, IPH6, and IPH7 (Figures 4D–G, *P* < 0.05). In addition, the level of *PPARGC1A* in IPH3 was the lowest compared to other cybrids (Figure 4H, *P* < 0.01). Meanwhile, the expressions of *TFB2M*, *NRF1*, and *PPARGC1A* in fibroblasts and *TFB1M* among cybrids presented poor differences (data not shown).

**FIGURE 5:**
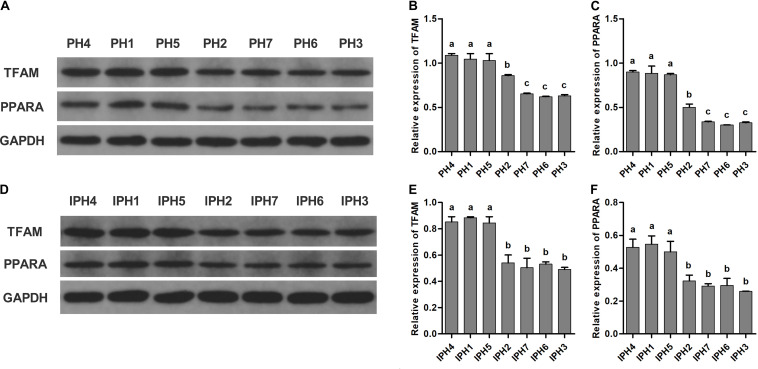
Measurements of PPARA and TFAM in fibroblasts and cybrids by western blot assays. **(A,D)** Illustrations of western blotting images in fibroblasts and cybrids; **(B,C)** protein levels of TFAM and PPARA in fibroblasts; **(E,F)** protein contents of TFAM and PPARA in cybrids. Different letters on columns meant significant differences at *P* < 0.05 levels. PH1-7, fibroblast with mitotype 1–7, respectively; IPH1-7, cybrid with mitotype 1–7, respectively.

For measurements of TFAM and PPARA, PH4, PH1, and PH5 reached the highest levels, followed by PH2, and the lowest appeared in PH7, PH6, and PH3 (Figures 5B,C, *P* < 0.05). For cybrids, IPH1, IPH4, and IPH5 synthesized larger TFAM and PPARA proteins than IPH2, IPH7, IPH6, and IPH3 (Figures 5E,F, *P* < 0.05).

### Expressions of Reproduction-Related Genes

**FIGURE 6:**
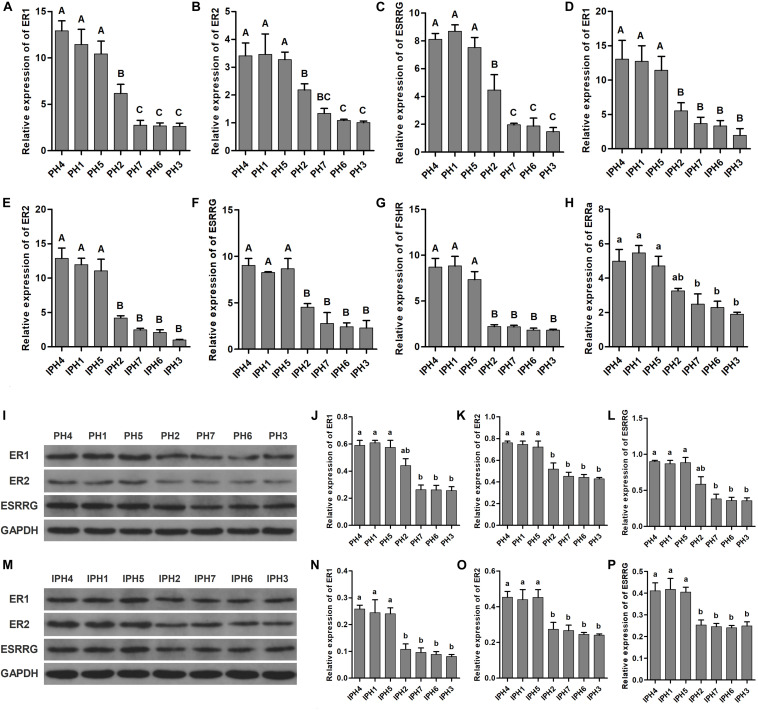
Expressions of reproduction-related genes in fibroblasts and cybrids. **(A–C)** Illustrated the mRNA expressions of *ER1*, *ER2*, and *ESRRG* in fibroblasts. **(D–H)** Presented the mRNA expressions of *ER1*, *ER2*, *ESRRG*, *FSHR*, and *ESRRa* in cybrids. **(I,M)** Illustrations of western blotting images in fibroblasts and cybrids. **(J–L)** Protein levels of ER1, ER2, and ESRRG in fibroblasts. **(N–P)** Protein contents of ER1, ER2, and ESRRG in cybrids. Different capital and lowercase letters on columns meant significant differences at *P* < 0.01 or *P* < 0.05, respectively. PH1-7, fibroblast with mitotype 1–7, respectively; IPH1-7, cybrid with mitotype 1–7, respectively.

Five reproduction-related genes (*ER1*, *ER2*, *ESRRG*, *FSHR*, and *ESRRa*) were measured for expression abundance in fibroblasts and cybrids by RT-qPCR (Figure 6). For fibroblasts, PH1, PH4, and PH5 owned the highest level on *ER1* and *ESRRG*, followed by PH2, and the last class was PH3, PH6, and PH7 (Figures 6A,C, *P* < 0.01). On *ER2*, PH1, PH4, and PH5 illustrated significantly higher levels than PH2, PH3, PH6, and PH7, besides, PH3 and PH6 exhibited lower expressions compared to PH2 (Figure 6B, *P* < 0.01). However, no statistical differences observed on *FSHR* and *ESRRa* among fibroblasts (data not shown). For cybrids, IPH1, IPH4 and IPH5 showed higher abundances of *ER1*, *ER2*, *ESRRG*, and *FSHR* than IPH2, IPH3, IPH6, and IPH7 (Figures 6D–G, *P* < 0.01), while IPH3, IPH6 and IPH7 presented lower *ESRRa* expressions compared to IPH1, IPH4, and IPH5 (Figure 6H, *P* < 0.05).

For protein analysis, PH1, PH4, and PH5 presented higher expressions of ER1 and ESRRG than PH3, PH6, and PH7 (Figures 6J,L, *P* < 0.05), and PH1, PH4 and PH5 owned greater ER2 contents than other fibroblasts (Figure 6K, *P* < 0.05). For cybrids, IPH1, IPH4, and IPH5 had significantly higher measurements on ER1, ER2 and ESRRG than IPH2, IPH3, IPH6, and IPH7 (Figures 6N–P, *P* < 0.05).

## Discussion

Due to porcine litter size is considered a trait with low heritability, DNA markers could be applied in molecular breeding for greater predictability on reproduction (Shields et al., 1985; King et al., 2003; Wang et al., 2018; Liu et al., 2019a). The potential role of mtDNA impacting pig reproduction traits was postulated for several decades (Sun et al., 2001; El Shourbagy et al., 2006; Wang et al., 2018), and the question remains open with some ambiguity in accumulated studies of mtDNA variations and associated reports.

In this study, association analysis advanced maximum 1.73 or 1.07 piglet-effects between mitotypes or mitogroups, respectively (Tables 1, 2, *P* < 0.05), which recurred in previous studies on oocyte number (Liu et al., 2019a) and litter size of born alive (Wang et al., 2018). HG1 has significantly higher piglets than HG2, and HG1 members (H1, H4, and H5) are generally prolific compared to HG2 members (H2, H3, H6, and H7).

Due to confounding variations, including nuclear genome, epigenetic phenomena and environmental factors, it is difficult to evaluate the mitochondrial genetic effects on animal traits directly. The transmitochondrial cybrid model is created by fusing cells devoid of mtDNA (ρ0 cells) with cytoplasts (enucleated cells) from different individuals, so the resultant cybrids have uniform nuclear background but different mitochondrial genome (Yu et al., 2015; Swerdlow et al., 2017), which are valid tools for dissecting mtDNA effect of dynamic biological function under the same background. Thus, cybrid model was utilized in this study to identify the correlations to cell metabolisms and gene expressions caused by mitotypes. It is noteworthy that prolific mitotypes (H1, H4, and H5) exhibited higher levels of OCRs, mtDNA copy numbers and transcriptions, expressions of mitochondrial biogenesis-related genes and reproduction-related genes, and lower ROS contents compared to common mitotypes (H2, H3, H6, and H7), which were consistent with the effect on litter size (Figures 2–5 and Table 1).

Optimal energy is necessary for oogenesis, fertilization and embryo development, which have strong impacts on mammalian reproduction performances (Sun et al., 2001; Santos et al., 2006; Amaral et al., 2013; Babayev and Seli, 2015). Compared to common mitotypes (H2, H3, H6, and H7), fibroblasts containing prolific mitotypes (H1, H4, and H5) exhibited generally higher OCRs, with an increase in basal respiration, ATP production, maximal respirations and spare respiratory capacity (Figure 2), reflecting an active energy metabolism and potential capability for greater reproductive characters.

Copy number and transcription of mitochondrial genome are dynamic and cell-specific biomarkers (Moraes, 2001), which depends on the regulation of mitochondrial biogenesis (Lee et al., 2015; Laubenthal et al., 2016) and ATP generation through oxidative phosphorylation (Wai et al., 2010). In the present research, cells with prolific mitotypes (H1, H4, and H5) contained larger mtDNA copy numbers and transcriptions than common mitotypes, which were consistent with OCR results. Previous studies reported that mitochondrial genome did not replicate at the early stage of embryonic development, and high-quality mtDNA copies were essential for cell division and energy supply (Sun et al., 2001; Babayev and Seli, 2015), which reflects the correlation of mtDNA copies, mitotypes and pig litter size.

A broad range of interactions occurred between nucleus and mitochondria, including not only constructing mitochondrial complexes, but also proteins or polypeptides of target DNA or RNA, which required for regulating gene replication, expression and epigenetic modification (Goffart and Wiesner, 2003; Kaniak-Golik and Skoneczna, 2015; Dunham-Snary et al., 2018). *TFAM*, *TFB1M*, *TFB2M*, *PPARA*, *NRF1*, and *PPARGC1A* are key members of nuclear-encoded mitochondrial genes, which involved in regulating mitochondrial biogenesis and energy metabolism (Choi et al., 2011; Li et al., 2016a, 2017). *ER1*, *ER2*, *ESRRG*, *FSHR*, and *ESRRa* are reproduction-related genes, which could receive hormone stimulation and trigger signaling cascades (Ahmed and Fisher, 2009; Choi et al., 2011). In addition, *ER1*, *ER2*, and *ESRRG* are key elements involved in the regulation of mitochondrial biogenesis, anti-oxidative stress, OXPHOS and anti-apoptosis (Wilson et al., 2002; Solakidi et al., 2005; Hulas-Stasiak and Gawron, 2007; Mehta et al., 2014; Fan et al., 2018). In this study, based on different levels of OCRs, mtDNA copy numbers or transcriptions in cybrids (Figure 3), prolific mitotypes (H1, H4, and H5) showed higher mRNA and protein levels of mitochondrial biogenesis-related genes (*TFAM* and *PPARA*) (Figures 4, 5) and reproduction-related genes (*ER1*, *ER2*, and *ESRRG*) (Figure 6), illustrating the outstanding ATP supply and potential competence for litter size.

Primary fibroblast cells, which interacted with nucleus interferences, still manifested cybrids’ profiles, i.e., prolific mitotypes (H1, H4, and H5) had higher levels of energy metabolism, mtDNA copies and gene expressions than common mitotypes (H2, H3, H6, and H7) (Figures 2–6), reflecting mtDNA effects on pig litter size.

The proliferation rate is a fundamental indicator for assessing cell states (Golias et al., 2004), which is a prerequisite for further experiments. In the current study, cell proliferation rates of fibroblasts or cybrids presented stable and similar growing states (Figures 2A,B), indicating the validity of verification experiments.

On the whole, these results presented that mitotypes associated with pig litter size, and cybrid models verified the mitotype effect by mitochondrial metabolic and gene expressional characters.

## Conclusion

This is the first report, to our knowledge, that demonstrates mitotype effect on pig TNBs, which ultimately confers differences on mitochondrial energy metabolic traits and gene expressions involved in mitochondrial biogenesis and reproduction traits using transmitochondrial cell models. This study provides an insight into mitotype effect on reproduction traits of domestic animals. Therefore, mitotype can be considered as candidate markers for animal selection and breeding.

## Data Availability Statement

The raw data supporting the conclusions of this article will be made available by the authors, without undue reservation.

## Ethics Statement

The animal study was reviewed and approved by the Experimental Animal Care and Use Committee of CAU.

## Author Contributions

XZo and HL conceived and designed the study. HL, JW, DW, and XZn performed the experiments. HL, DW, and CN analyzed the data. XZg, HL, JX, and MK collected the samples. XZo and HL interpreted the data and drafted the manuscript. All authors read and approved the final manuscript.

## Conflict of Interest

The authors declare that the research was conducted in the absence of any commercial or financial relationships that could be construed as a potential conflict of interest.

## Footnotes

1 http://www.megasoftware.net/

2 http://users-birc.au.dk/biopv/php/fabox/

3 http://www.fluxus-engineering.com/sharenet.htm

## Supplementary Material

Supplementary Figure 1 Oxygen consumption rates for different cell lines. **(A–D)** Indicated basal respirations, maximal respirations, ATP productions and spare respiratory capacities in fibroblasts. **(E–H)** Represented basal respirations, maximal respirations, ATP productions and spare respiratory capacities in cybrids. Different letters on columns meant significant differences at *P* < 0.01. PH1-7, fibroblast with mitotype 1–7, respectively; IPH1-7, cybrid with mitotype 1–7, respectively.

Supplementary Table 1 Primers for detections of mitochondrial genome sequences, mtDNA copy number, and mRNA expressions.

Supplementary Table 2 Polymorphic sites detected among mitotypes.

Supplementary Table 3 Polymorphic sites for identifying mitogroups.
