# Supplementary figures and images for: Cybrid Model Supports Mitochondrial Genetic Effect on Pig Litter Size
Source: Front Genet. 2020 Dec 15;11:579382. doi: 10.3389/fgene.2020.579382 (PMC7770168; doi:10.3389/fgene.2020.579382)

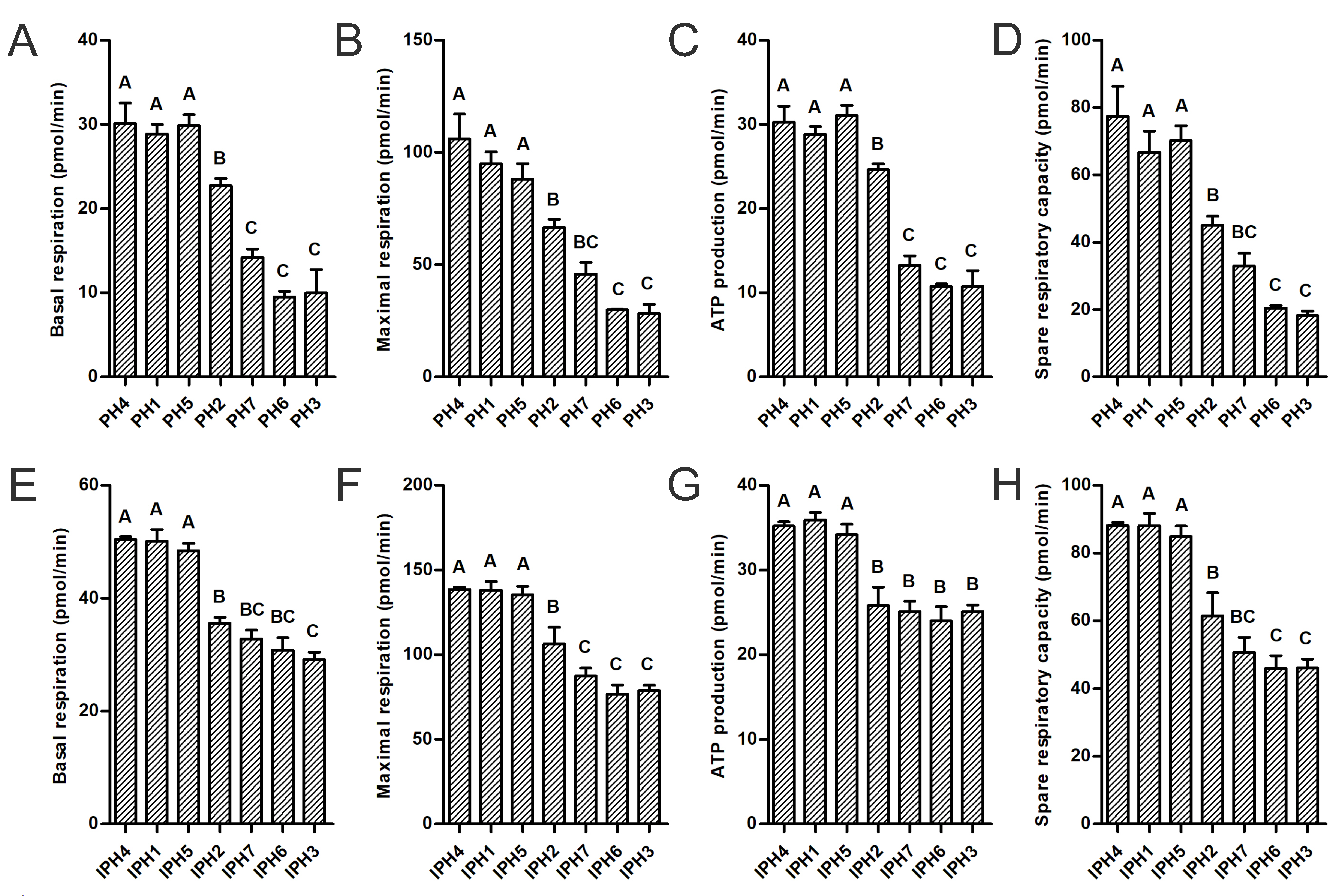

Supplement: Supplementary Figure 1 — Oxygen consumption rates for different cell lines. (A–D) Indicated basal respirations, maximal respirations, ATP productions and spare respiratory capacities in fibroblasts. (E–H) Represented basal respirations, maximal respirations, ATP productions and spare respiratory capacities in cybrids. Different letters on columns meant significant differences at P < 0.01. PH1-7, fibroblast with mitotype 1–7, respectively; IPH1-7, cybrid with mitotype 1–7, respectively. [file Image_1.TIF]
