# Supplementary material for: Cybrid Model Supports Mitochondrial Genetic Effect on Pig Litter Size
Source: Front Genet. 2020 Dec 15;11:579382. doi: 10.3389/fgene.2020.579382 (PMC7770168; doi:10.3389/fgene.2020.579382)
Supplement: Supplementary Table 1 — Primers for detections of mitochondrial genome sequences, mtDNA copy number, and mRNA expressions. [file Table_1.DOCX]

Table S1. Primers for detections of mitochondrial genome sequences, mtDNA copy number, and mRNA expressions.

| Target | Sequence (5'→3') | Length (bp) | Tm (°C) |
| --- | --- | --- | --- |
| mitochondrial genome sequencing | | | |
| MGS1 | F: CCGATAGACCTTACCAACCCTTG | 5737 | 60 |
|  | R: GTGAATAGGAAGATGAAGCCCAG |  |  |
| MGS2 | F: ATAACACCAGCCCTCTCGTC | 7276 | 60 |
|  | R: CTACGCCTTCTCATCCGATA |  |  |
| MGS3 | F: GACATTGAATAACCCTACAGACCG | 5689 | 60 |
|  | R: GGTAATCCAAGCACACTTTCCAG |  |  |
| mtDNA copy number | | | |
| Beta-globin | F: AGCCAGCAGCCACCTACATT | 144 | 60 |
|  | R: CCACCAACTTCGTCCACATTCA |  |  |
| mtDNA | F: ACCTACTAGGAGACCCAGACAACT | 107 | 60 |
|  | R: TGAACGTAGGATAGCGTAGGCGAA |  |  |
| mRNA expression | | | |
| GAPDH | F: ATTCCACCCACGGCAAGTTC | 260 | 52-60 |
|  | R: GTTCACGCCCATCACAAACA |  |  |
| mtRNA | F: ACGCCCACAATCTGAA | 212 | 58 |
|  | R: GCTTGATACCTGCTCCTTT |  |  |
| NRF1 | F: ACCATCCAGACAACGCAA | 230 | 55 |
|  | R: ACTCCAGTAAGTGCTCCGAC |  |  |
| PPARA | F: CCAGTATTGTCGTTTCCACA | 166 | 55 |
|  | R: CTTGGCGAGAGACTTGAGA |  |  |
| TFAM | F: TGTGCGGTTTGTGGAAGT | 263 | 55 |
|  | R: ACCTGCCAGTCTGCCCTAT |  |  |
| TFB1M | F: CGAGGGCTTGGAATGTTA | 204 | 55 |
|  | R: CGTGTGCCTGAGTTCTTCT |  |  |
| TFB2M | F: GCAAGGAGGAAGGATGTT | 243 | 55 |
|  | R: CAAGTAATGCTCGTGTCAGG |  |  |
| PPARGC1A | F: GCTATGGTTTCATTACCTACCG | 271 | 58 |
|  | R: ATCCTCAGCCAGGGAACAT |  |  |
| FSHR | F: TGGCAGAAGATGGTGAGTC | 273 | 56 |
|  | R: CCAGATTACACATAAGGAACCG |  |  |
| ESRRA | F: ACCGAGAGATTGTGGTCAC | 283 | 56 |
|  | R: AACATACTCCTCACGCTCC |  |  |
| ESRRG | F: TCTTCCTTCTTCCTCGCTT | 168 | 55 |
|  | R: TTGATTCCTTAGTCACTGGG |  |  |
| ER1 | F: CACACTAAGAAGAACAGCCC | 283 | 58 |
|  | R: GCCAGACAAGACCAATCA |  |  |
| ER2 | F: TATCTCCTCCCAGCAGCAGTCT | 153 | 56 |
|  | R: TTCAGCATCTCCAGCAGCA |  |  |
