# Supplementary material for: Cybrid Model Supports Mitochondrial Genetic Effect on Pig Litter Size
Source: Front Genet. 2020 Dec 15;11:579382. doi: 10.3389/fgene.2020.579382 (PMC7770168; doi:10.3389/fgene.2020.579382)
Supplement: Supplementary Table 2 — Polymorphic sites detected among mitotypes. [file Table_2.DOCX]

Table S2. Polymorphic sites detected among mitotypes.

| Number | Site (bp) | Gene | NC_000845.1 | Haplotype | | | | | | |
| --- | --- | --- | --- | --- | --- | --- | --- | --- | --- | --- |
|  |  |  |  | H1 | H2 | H3 | H4 | H5 | H6 | H7 |
| 1 | 109 | D-loop | T | C | T | T | C | C | T | T |
| 2 | 124 | D-loop | T | A | T | T | A | A | T | T |
| 3 | 131 | D-loop | G | A | G | G | A | A | G | G |
| 4 | 136 | D-loop | C | - | C | C | - | - | C | C |
| 5 | 145 | D-loop | C | T | C | C | T | T | C | C |
| 6 | 153 | D-loop | C | T | C | C | T | T | C | C |
| 7 | 158 | D-loop | A | G | A | A | G | G | A | A |
| 8 | 181 | D-loop | T | C | C | T | C | C | T | T |
| 9 | 241 | D-loop | T | C | T | T | C | T | T | T |
| 10 | 279 | D-loop | C | C | C | C | T | T | C | T |
| 11 | 294 | D-loop | A | G | A | A | G | G | A | A |
| 12 | 306 | D-loop | C | T | C | C | T | T | C | C |
| 13 | 323 | D-loop | C | T | C | C | T | T | C | C |
| 14 | 390 | D-loop | C | T | C | C | T | T | C | C |
| 15 | 405 | D-loop | T | C | T | T | T | T | C | T |
| 16 | 452 | D-loop | C | T | C | C | C | C | C | C |
| 17 | 474 | D-loop | C | C | C | C | C | T | C | C |
| 18 | 575 | D-loop | A | G | A | A | G | G | A | A |
| 19 | 992 | D-loop | T | T | T | T | T | T | C | T |
| 20 | 1013-1014 | D-loop | - | CTTATAAAACA | - | - | - | - | - | - |
| 21 | 1089 | D-loop | T | C | T | T | C | C | T | T |
| 22 | 1096 | D-loop | A | G | A | A | G | G | A | A |
| 23 | 1146 | D-loop | T | C | T | T | C | C | T | T |
| 24 | 1225 | tRNA-Phe | A | G | A | A | G | G | A | A |
| 25 | 1234 | tRNA-Phe | G | A | G | G | G | G | G | G |
| 26 | 1236 | tRNA-Phe | T | C | T | T | C | C | T | T |
| 27 | 1333 | rRNA_12S | T | C | T | T | C | C | T | T |
| 28 | 1550-1551 | rRNA_12S | - | A | A | - | - | A | A | - |
| 29 | 1559 | rRNA_12S | T | C | T | T | C | C | T | T |
| 30 | 1910 | rRNA_12S | G | A | G | G | A | A | G | G |
| 31 | 1984 | rRNA_12S | T | C | T | T | C | C | T | T |
| 32 | 1991 | rRNA_12S | C | T | C | C | T | T | C | C |
| 33 | 2255 | tRNA-Val | C | T | C | C | T | T | C | C |
| 34 | 2259 | tRNA-Val | G | A | G | G | A | A | G | G |
| 35 | 2294 | rRNA_16S | C | T | C | C | T | T | C | C |
| 36 | 2534 | rRNA_16S | C | T | C | C | T | T | C | C |
| 37 | 2679 | rRNA_16S | T | C | C | C | C | T | C | C |
| 38 | 2985 | rRNA_16S | C | T | C | C | T | T | C | C |
| 39 | 3009 | rRNA_16S | A | G | A | A | G | G | A | A |
| 40 | 3023 | rRNA_16S | C | T | C | C | T | T | C | C |
| 41 | 3287 | rRNA_16S | A | G | A | A | G | G | A | A |
| 42 | 3355 | rRNA_16S | T | C | T | T | T | T | T | T |
| 43 | 3372 | rRNA_16S | C | T | C | C | T | T | C | C |
| 44 | 3561 | rRNA_16S | G | A | G | G | A | A | G | G |
| 45 | 3794 | rRNA_16S | T | A | T | T | A | A | T | T |
| 46 | 3920 | - | A | G | A | A | G | G | A | A |
| 47 | 3936 | ND1 | T | C | T | T | C | C | T | T |
| 48 | 3951 | ND1 | T | C | T | T | C | C | T | T |
| 49 | 4002 | ND1 | G | A | G | G | A | A | G | G |
| 50 | 4237 | ND1 | C | C | T | C | C | C | C | C |
| 51 | 4263 | ND1 | C | T | C | C | T | T | C | C |
| 52 | 4290 | ND1 | C | T | C | C | T | T | C | C |
| 53 | 4341 | ND1 | C | T | C | C | T | T | C | C |
| 54 | 4359 | ND1 | A | G | A | A | G | G | A | A |
| 55 | 4380 | ND1 | A | G | A | A | G | G | A | A |
| 56 | 4392 | ND1 | T | T | T | C | T | T | C | C |
| 57 | 4410 | ND1 | A | C | A | A | C | C | A | A |
| 58 | 4632 | ND1 | T | C | T | T | C | C | T | T |
| 59 | 4658 | ND1 | C | T | C | C | T | T | C | C |
| 60 | 4675 | ND1 | C | T | C | C | T | T | C | C |
| 61 | 4767 | ND1 | C | T | C | C | T | T | C | C |
| 62 | 4860 | ND1 | C | T | C | C | T | T | C | C |
| 63 | 5089 | ND2 | T | A | T | T | A | A | T | T |
| 64 | 5128 | ND2 | G | A | G | G | A | A | G | G |
| 65 | 5290 | ND2 | A | G | A | A | G | G | A | A |
| 66 | 5384 | ND2 | A | C | A | A | C | C | A | A |
| 67 | 5392 | ND2 | A | A | A | A | G | A | A | A |
| 68 | 5473 | ND2 | G | A | G | G | A | A | G | G |
| 69 | 5549 | ND2 | C | T | C | C | T | T | C | C |
| 70 | 5557 | ND2 | A | G | A | A | G | G | A | A |
| 71 | 5593 | ND2 | G | A | G | G | A | A | G | G |
| 72 | 5599 | ND2 | T | C | T | T | C | C | T | T |
| 73 | 5629 | ND2 | C | T | C | C | T | T | C | C |
| 74 | 5674 | ND2 | C | T | C | C | T | T | C | C |
| 75 | 5718 | ND2 | T | C | T | T | C | C | T | T |
| 76 | 5794 | ND2 | A | G | A | A | G | G | A | A |
| 77 | 5801 | ND2 | G | A | G | G | A | A | G | G |
| 78 | 5869 | ND2 | G | A | G | G | A | A | G | G |
| 79 | 5884 | ND2 | T | C | T | T | C | C | T | T |
| 80 | 5998 | ND2 | G | G | A | G | G | G | G | G |
| 81 | 6013 | ND2 | C | T | C | C | T | T | C | C |
| 82 | 6059 | ND2 | T | C | T | T | C | C | T | T |
| 83 | 6074 | ND2 | A | A | G | A | A | A | A | A |
| 84 | 6085 | ND2 | A | G | A | A | G | G | A | A |
| 85 | 6092 | ND2 | G | A | G | G | A | A | G | G |
| 86 | 6217 | tRNA-Ala | C | T | C | C | T | T | C | C |
| 87 | 6219 | tRNA-Ala | T | C | T | T | C | C | T | T |
| 88 | 6429 | tRNA-Cys | T | C | T | T | C | C | T | T |
| 89 | 6708 | COX1 | C | C | C | C | C | C | C | T |
| 90 | 6738 | COX1 | G | G | G | G | G | G | A | G |
| 91 | 6846 | COX1 | T | A | T | T | A | A | T | T |
| 92 | 6873 | COX1 | A | G | A | A | G | G | A | A |
| 93 | 6891 | COX1 | T | C | T | T | C | C | T | T |
| 94 | 6909 | COX1 | T | C | T | T | T | T | T | T |
| 95 | 6930 | COX1 | G | A | G | G | A | A | G | G |
| 96 | 6943 | COX1 | T | C | T | T | C | C | T | T |
| 97 | 7029 | COX1 | C | T | C | C | T | T | C | C |
| 98 | 7242 | COX1 | C | T | C | C | T | T | C | C |
| 99 | 7260 | COX1 | A | G | A | A | G | G | A | A |
| 100 | 7368 | COX1 | T | C | T | T | C | C | T | T |
| 101 | 7407 | COX1 | A | G | A | A | G | G | A | A |
| 102 | 7434 | COX1 | T | C | T | T | C | C | T | T |
| 103 | 7590 | COX1 | T | C | T | T | C | C | T | T |
| 104 | 7671 | COX1 | C | T | C | C | T | T | C | C |
| 105 | 7758 | COX1 | C | T | C | C | T | T | C | C |
| 106 | 7938 | COX1 | C | T | C | C | T | T | C | C |
| 107 | 8188 | tRNA-Asp | G | A | G | G | G | G | G | G |
| 108 | 8292 | COX2 | C | T | C | C | T | T | C | C |
| 109 | 8334 | COX2 | C | T | C | C | T | T | C | C |
| 110 | 8419 | COX2 | C | T | C | C | T | T | C | C |
| 111 | 8466 | COX2 | C | T | C | C | C | C | C | C |
| 112 | 8526 | COX2 | C | T | C | C | T | T | C | C |
| 113 | 8634 | COX2 | A | G | A | A | G | G | A | A |
| 114 | 8664 | COX2 | A | G | A | A | G | G | A | A |
| 115 | 8682 | COX2 | G | A | G | G | A | A | G | G |
| 116 | 8694 | COX2 | C | T | C | C | T | T | C | C |
| 117 | 8979 | ATP8 | C | T | C | C | T | T | C | C |
| 118 | 9077 | ATP8 | T | C | T | T | C | C | T | T |
| 119 | 9078 | ATP8 | T | C | T | T | C | C | T | T |
| 120 | 9146 | ATP8 | T | C | T | T | C | C | T | T |
| 121 | 9155 | ATP8 | C | T | C | C | T | T | C | C |
| 122 | 9289 | ATP6 | T | T | T | T | T | C | T | T |
| 123 | 9293 | ATP6 | A | G | A | A | A | A | A | A |
| 124 | 9333 | ATP6 | C | C | C | C | C | C | C | T |
| 125 | 9356 | ATP6 | C | T | C | C | T | T | C | C |
| 126 | 9474 | ATP6 | T | C | T | C | C | C | T | T |
| 127 | 9526 | ATP6 | T | C | T | T | C | C | T | T |
| 128 | 9673 | ATP6 | A | G | A | A | G | G | A | A |
| 129 | 9710 | ATP6 | T | C | T | T | C | C | T | T |
| 130 | 9894 | COX3 | C | T | C | C | T | T | C | C |
| 131 | 9991 | COX3 | G | A | G | G | A | A | G | G |
| 132 | 10021 | COX3 | T | C | T | T | C | C | T | T |
| 133 | 10405 | COX3 | G | A | G | G | A | A | G | G |
| 134 | 10450 | COX3 | A | G | A | A | G | G | A | A |
| 135 | 10601 | tRNA-Gly | T | C | T | T | C | C | T | T |
| 136 | 10674 | ND3 | C | T | C | C | T | T | C | C |
| 137 | 10737 | ND3 | G | A | G | G | A | A | G | G |
| 138 | 10865 | ND3 | T | C | T | T | C | C | T | T |
| 139 | 10939 | ND3 | T | C | T | T | C | C | T | T |
| 140 | 10992 | ND3 | G | A | G | G | A | A | G | G |
| 141 | 11083 | ND4L | T | C | T | T | C | C | T | T |
| 142 | 11105 | ND4L | G | A | G | G | A | A | G | G |
| 143 | 11110 | ND4L | C | T | C | C | T | T | C | C |
| 144 | 11180 | ND4L | C | T | C | C | T | T | C | C |
| 145 | 11210 | ND4L | A | G | A | A | G | G | A | A |
| 146 | 11248 | ND4L | C | C | C | C | C | C | C | T |
| 147 | 11287 | ND4L | A | G | A | A | G | G | A | A |
| 148 | 11293 | ND4L | G | A | G | G | A | A | G | G |
| 149 | 11353 | ND4L | T | C | T | T | C | C | T | T |
| 150 | 11604 | ND4 | T | C | T | T | C | C | T | T |
| 151 | 11707 | ND4 | C | T | C | C | T | T | C | C |
| 152 | 11751 | ND4 | T | C | T | T | C | C | T | T |
| 153 | 11806 | ND4 | C | C | T | C | C | C | C | C |
| 154 | 11865 | ND4 | T | C | T | T | C | C | T | T |
| 155 | 11985 | ND4 | G | A | G | G | A | A | G | G |
| 156 | 12030 | ND4 | C | T | C | C | T | T | C | C |
| 157 | 12162 | ND4 | C | T | C | C | T | T | C | C |
| 158 | 12219 | ND4 | C | T | C | C | T | T | C | C |
| 159 | 12276 | ND4 | T | C | T | C | C | C | C | C |
| 160 | 12291 | ND4 | G | A | G | G | A | A | G | G |
| 161 | 12390 | ND4 | G | A | G | G | A | A | G | G |
| 162 | 12439 | ND4 | A | A | G | G | A | A | G | G |
| 163 | 12504 | ND4 | C | T | C | C | T | T | C | C |
| 164 | 12570 | ND4 | C | T | C | C | T | T | C | C |
| 165 | 12596 | ND4 | C | T | C | C | T | T | C | C |
| 166 | 12879 | tRNA-Leu | A | G | A | A | G | G | A | A |
| 167 | 12883 | tRNA-Leu | C | T | C | C | T | T | C | C |
| 168 | 12970 | ND5 | A | G | A | A | G | G | A | A |
| 169 | 13034 | ND5 | A | A | A | A | A | A | A | G |
| 170 | 13354 | ND5 | A | C | A | A | C | C | A | A |
| 171 | 13393 | ND5 | G | C | G | G | G | G | G | G |
| 172 | 13399 | ND5 | T | C | T | T | C | C | T | T |
| 173 | 13502 | ND5 | C | T | C | C | T | T | C | C |
| 174 | 13526 | ND5 | C | T | C | C | T | T | C | C |
| 175 | 13759 | ND5 | T | C | T | T | C | C | T | T |
| 176 | 13918 | ND5 | C | T | C | C | T | T | C | C |
| 177 | 14119 | ND5 | C | C | C | C | T | T | C | C |
| 178 | 14130 | ND5 | T | C | T | T | C | C | T | T |
| 179 | 14134 | ND5 | T | C | T | T | C | C | T | T |
| 180 | 14218 | ND5 | C | C | C | C | C | A | C | C |
| 181 | 14233 | ND5 | G | G | G | G | A | G | G | G |
| 182 | 14234 | ND5 | A | C | A | A | C | C | A | A |
| 183 | 14320 | ND5 | T | C | T | T | C | C | T | T |
| 184 | 14482 | ND5 | C | A | C | C | A | A | C | C |
| 185 | 14560 | ND5 | A | G | A | A | G | G | A | A |
| 186 | 14601 | ND5 | T | T | C | T | T | T | T | T |
| 187 | 14628 | ND5 | C | C | C | C | C | C | T | C |
| 188 | 14659 | ND5 | C | T | C | C | T | T | C | C |
| 189 | 14733 | ND5 | C | T | C | C | T | T | C | C |
| 190 | 14760 | ND6 | T | C | T | T | C | C | T | T |
| 191 | 14868 | ND6 | C | T | C | C | T | T | C | C |
| 192 | 14919 | ND6 | G | A | G | G | A | A | G | G |
| 193 | 14946 | ND6 | C | T | C | C | T | T | C | C |
| 194 | 15030 | ND6 | A | G | A | A | G | G | A | A |
| 195 | 15048 | ND6 | A | G | A | A | G | G | A | A |
| 196 | 15072 | ND6 | C | T | C | C | T | T | C | C |
| 197 | 15141 | ND6 | A | A | A | A | G | G | A | A |
| 198 | 15198 | ND6 | C | T | C | C | T | T | C | C |
| 199 | 15258 | ND6 | C | T | C | C | T | T | C | C |
| 200 | 15283 | tRNA-Glu | T | C | T | T | C | C | T | T |
| 201 | 15527 | CYTB | T | T | T | T | T | T | C | T |
| 202 | 15548 | CYTB | T | C | T | T | C | C | T | T |
| 203 | 15584 | CYTB | T | C | T | T | C | C | T | T |
| 204 | 15608 | CYTB | A | G | A | A | G | G | A | A |
| 205 | 15644 | CYTB | T | C | T | T | C | C | T | T |
| 206 | 15695 | CYTB | C | C | C | C | T | C | C | C |
| 207 | 15884 | CYTB | C | T | C | C | T | T | C | C |
| 208 | 16034 | CYTB | A | G | A | A | G | G | A | A |
| 209 | 16181 | CYTB | T | C | T | T | C | C | T | T |
| 210 | 16215 | CYTB | T | C | T | T | C | C | T | T |
| 211 | 16217 | CYTB | G | A | G | G | A | A | G | G |
| 212 | 16220 | CYTB | C | T | C | C | T | T | C | C |
| 213 | 16224 | CYTB | G | A | G | G | A | A | G | G |
| 214 | 16281 | CYTB | A | A | G | G | A | A | G | G |
| 215 | 16340 | CYTB | T | T | T | T | T | T | C | T |
| 216 | 16379 | CYTB | G | A | G | G | A | A | G | G |
| 217 | 16415 | CYTB | C | T | C | C | T | T | C | C |
| 218 | 16475 | CYTB | A | A | A | A | G | G | A | A |
| 219 | 16487 | tRNA-Thr | C | T | C | C | T | T | C | C |
| 220 | 16531 | tRNA-Thr | G | A | G | G | A | A | G | G |
