# Supplementary material for: Cybrid Model Supports Mitochondrial Genetic Effect on Pig Litter Size
Source: Front Genet. 2020 Dec 15;11:579382. doi: 10.3389/fgene.2020.579382 (PMC7770168; doi:10.3389/fgene.2020.579382)
Supplement: Supplementary Table 3 — Polymorphic sites for identifying mitogroups. [file Table_3.DOCX]

Table S3. Polymorphic sites for identifying mitogroups.

| Site (bp) | Gene | HG1 | HG2 | Variation Type |
| --- | --- | --- | --- | --- |
| 109 | D-loop | T | C | — |
| 124 | D-loop | T | A | — |
| 131 | D-loop | G | A | — |
| 136 | D-loop | C | - | — |
| 145 | D-loop | C | T | — |
| 153 | D-loop | C | T | — |
| 158 | D-loop | A | G | — |
| 294 | D-loop | A | G | — |
| 306 | D-loop | C | T | — |
| 323 | D-loop | C | T | — |
| 390 | D-loop | C | T | — |
| 575 | D-loop | A | G | — |
| 1089 | D-loop | T | C | — |
| 1096 | D-loop | A | G | — |
| 1146 | D-loop | T | C | — |
| 1225 | tRNA-Phe | A | G | Missense mutation |
| 1236 | tRNA-Phe | T | C | Missense mutation |
| 1333 | rRNA_12S | T | C | Missense mutation |
| 1559 | rRNA_12S | T | C | Missense mutation |
| 1910 | rRNA_12S | G | A | Missense mutation |
| 1984 | rRNA_12S | T | C | Missense mutation |
| 1991 | rRNA_12S | C | T | Missense mutation |
| 2255 | tRNA-Val | C | T | Missense mutation |
| 2259 | tRNA-Val | G | A | Missense mutation |
| 2294 | rRNA_16S | C | T | Missense mutation |
| 2534 | rRNA_16S | C | T | Missense mutation |
| 2985 | rRNA_16S | C | T | Missense mutation |
| 3009 | rRNA_16S | A | G | Missense mutation |
| 3023 | rRNA_16S | C | T | Missense mutation |
| 3287 | rRNA_16S | A | G | Missense mutation |
| 3372 | rRNA_16S | C | T | Missense mutation |
| 3561 | rRNA_16S | G | A | Missense mutation |
| 3794 | rRNA_16S | T | A | Missense mutation |
| 3920 | - | A | G | — |
| 3936 | ND1 | T | C | Synonymous mutation |
| 3951 | ND1 | T | C | Synonymous mutation |
| 4002 | ND1 | G | A | Synonymous mutation |
| 4263 | ND1 | C | T | Synonymous mutation |
| 4290 | ND1 | C | T | Synonymous mutation |
| 4341 | ND1 | C | T | Synonymous mutation |
| 4359 | ND1 | A | G | Synonymous mutation |
| 4380 | ND1 | A | G | Synonymous mutation |
| 4410 | ND1 | A | C | Synonymous mutation |
| 4632 | ND1 | T | C | Synonymous mutation |
| 4658 | ND1 | C | T | Missense mutation |
| 4675 | ND1 | C | T | Missense mutation |
| 4767 | ND1 | C | T | Synonymous mutation |
| 4860 | ND1 | C | T | Synonymous mutation |
| 5089 | ND2 | T | A | Synonymous mutation |
| 5128 | ND2 | G | A | Synonymous mutation |
| 5290 | ND2 | A | G | Synonymous mutation |
| 5384 | ND2 | A | C | Missense mutation |
| 5473 | ND2 | G | A | Synonymous mutation |
| 5549 | ND2 | C | T | Synonymous mutation |
| 5557 | ND2 | A | G | Synonymous mutation |
| 5593 | ND2 | G | A | Synonymous mutation |
| 5599 | ND2 | T | C | Synonymous mutation |
| 5629 | ND2 | C | T | Synonymous mutation |
| 5674 | ND2 | C | T | Synonymous mutation |
| 5718 | ND2 | T | C | Missense mutation |
| 5794 | ND2 | A | G | Synonymous mutation |
| 5801 | ND2 | G | A | Missense mutation |
| 5869 | ND2 | G | A | Synonymous mutation |
| 5884 | ND2 | T | C | Synonymous mutation |
| 6013 | ND2 | C | T | Synonymous mutation |
| 6059 | ND2 | T | C | Synonymous mutation |
| 6085 | ND2 | A | G | Synonymous mutation |
| 6092 | ND2 | G | A | Missense mutation |
| 6217 | tRNA-Ala | C | T | Missense mutation |
| 6219 | tRNA-Ala | T | C | Missense mutation |
| 6429 | tRNA-Cys | T | C | Missense mutation |
| 6846 | COX1 | T | A | Synonymous mutation |
| 6873 | COX1 | A | G | Synonymous mutation |
| 6891 | COX1 | T | C | Synonymous mutation |
| 6930 | COX1 | G | A | Synonymous mutation |
| 6943 | COX1 | T | C | Synonymous mutation |
| 7029 | COX1 | C | T | Synonymous mutation |
| 7242 | COX1 | C | T | Synonymous mutation |
| 7260 | COX1 | A | G | Synonymous mutation |
| 7368 | COX1 | T | C | Synonymous mutation |
| 7407 | COX1 | A | G | Synonymous mutation |
| 7434 | COX1 | T | C | Synonymous mutation |
| 7590 | COX1 | T | C | Synonymous mutation |
| 7671 | COX1 | C | T | Synonymous mutation |
| 7758 | COX1 | C | T | Synonymous mutation |
| 7938 | COX1 | C | T | Synonymous mutation |
| 8292 | COX2 | C | T | Synonymous mutation |
| 8334 | COX2 | C | T | Synonymous mutation |
| 8419 | COX2 | C | T | Synonymous mutation |
| 8526 | COX2 | C | T | Synonymous mutation |
| 8634 | COX2 | A | G | Synonymous mutation |
| 8664 | COX2 | A | G | Synonymous mutation |
| 8682 | COX2 | G | A | Synonymous mutation |
| 8694 | COX2 | C | T | Synonymous mutation |
| 8979 | MRC_V（ATP8） | C | T | Synonymous mutation |
| 9077 | MRC_V（ATP8） | T | C | Missense mutation |
| 9078 | MRC_V（ATP8） | T | C | Missense mutation |
| 9146 | MRC_V（ATP8） | T | C | Missense mutation |
| 9155 | MRC_V（ATP8） | C | T | Missense mutation |
| 9356 | MRC_V（ATP6） | C | T | Synonymous mutation |
| 9526 | MRC_V（ATP6） | T | C | Missense mutation |
| 9673 | MRC_V（ATP6） | A | G | Missense mutation |
| 9710 | MRC_V（ATP6） | T | C | Synonymous mutation |
| 9894 | COX3 | C | T | Missense mutation |
| 9991 | COX3 | G | A | Synonymous mutation |
| 10021 | COX3 | T | C | Synonymous mutation |
| 10405 | COX3 | G | A | Synonymous mutation |
| 10450 | COX3 | A | G | Synonymous mutation |
| 10601 | tRNA-Gly | T | C | Missense mutation |
| 10674 | ND3 | C | T | Missense mutation |
| 10737 | ND3 | G | A | Missense mutation |
| 10865 | ND3 | T | C | Synonymous mutation |
| 10939 | ND3 | T | C | Missense mutation |
| 10992 | ND3 | G | A | Missense mutation |
| 11083 | ND4L | T | C | Synonymous mutation |
| 11105 | ND4L | G | A | Missense mutation |
| 11110 | ND4L | C | T | Synonymous mutation |
| 11180 | ND4L | C | T | Synonymous mutation |
| 11210 | ND4L | A | G | Missense mutation |
| 11287 | ND4L | A | G | Synonymous mutation |
| 11293 | ND4L | G | A | Synonymous mutation |
| 11353 | ND4L | T | C | Synonymous mutation |
| 11604 | ND4 | T | C | Synonymous mutation |
| 11707 | ND4 | C | T | Synonymous mutation |
| 11751 | ND4 | T | C | Synonymous mutation |
| 11865 | ND4 | T | C | Synonymous mutation |
| 11985 | ND4 | G | A | Synonymous mutation |
| 12030 | ND4 | C | T | Synonymous mutation |
| 12162 | ND4 | C | T | Synonymous mutation |
| 12219 | ND4 | C | T | Synonymous mutation |
| 12291 | ND4 | G | A | Synonymous mutation |
| 12390 | ND4 | G | A | Synonymous mutation |
| 12439 | ND4 | G | A | Missense mutation |
| 12504 | ND4 | C | T | Synonymous mutation |
| 12570 | ND4 | C | T | Synonymous mutation |
| 12596 | ND4 | C | T | Missense mutation |
| 12879 | tRNA-Leu | A | G | Missense mutation |
| 12883 | tRNA-Leu | C | T | Missense mutation |
| 12970 | ND5 | A | G | Synonymous mutation |
| 13354 | ND5 | A | C | Synonymous mutation |
| 13399 | ND5 | T | C | Synonymous mutation |
| 13502 | ND5 | C | T | Synonymous mutation |
| 13526 | ND5 | C | T | Missense mutation |
| 13759 | ND5 | T | C | Synonymous mutation |
| 13918 | ND5 | C | T | Synonymous mutation |
| 14130 | ND5 | T | C | Missense mutation |
| 14134 | ND5 | T | C | Synonymous mutation |
| 14234 | ND5 | A | C | Missense mutation |
| 14320 | ND5 | T | C | Synonymous mutation |
| 14482 | ND5 | C | A | Synonymous mutation |
| 14560 | ND5 | A | G | Synonymous mutation |
| 14659 | ND5 | C | T | Synonymous mutation |
| 14733 | ND5 | C | T | Missense mutation |
| 14760 | ND6 | T | C | Synonymous mutation |
| 14868 | ND6 | C | T | Synonymous mutation |
| 14919 | ND6 | G | A | Synonymous mutation |
| 14946 | ND6 | C | T | Synonymous mutation |
| 15030 | ND6 | A | G | Synonymous mutation |
| 15048 | ND6 | A | G | Synonymous mutation |
| 15072 | ND6 | C | T | Synonymous mutation |
| 15198 | ND6 | C | T | Synonymous mutation |
| 15258 | ND6 | C | T | Synonymous mutation |
| 15283 | tRNA-Glu | T | C | Missense mutation |
| 15548 | CYTB | T | C | Synonymous mutation |
| 15584 | CYTB | T | C | Synonymous mutation |
| 15608 | CYTB | A | G | Synonymous mutation |
| 15644 | CYTB | T | C | Synonymous mutation |
| 15884 | CYTB | C | T | Synonymous mutation |
| 16034 | CYTB | A | G | Synonymous mutation |
| 16181 | CYTB | T | C | Synonymous mutation |
| 16215 | CYTB | T | C | Synonymous mutation |
| 16217 | CYTB | G | A | Synonymous mutation |
| 16220 | CYTB | C | T | Synonymous mutation |
| 16224 | CYTB | G | A | Missense mutation |
| 16281 | CYTB | G | A | Missense mutation |
| 16379 | CYTB | G | A | Synonymous mutation |
| 16415 | CYTB | C | T | Synonymous mutation |
| 16487 | tRNA-Thr | C | T | Missense mutation |
| 16531 | tRNA-Thr | G | A | Missense mutation |
